# A Study of Handling Cytotoxic Drugs and Risk of Birth Defects in Offspring of Female Veterinarians

**DOI:** 10.3390/ijerph110606216

**Published:** 2014-06-12

**Authors:** Adeleh Shirangi, Carol Bower, C. D’Arcy J. Holman, David B. Preen, Neville Bruce

**Affiliations:** 1 School of Population Health, The University of Western Australia, Crawley, WA 6009, Australia; E-Mails: darcy.holman@uwa.edu.au (C.D.J.H.); david.preen@uwa.edu.au (D.B.P.); 2 Department of Epidemiology, Population Sciences, Telethon Kids Institute, 100 Roberts Road, Subiaco 6008, Australia; E-Mail: carol.bower@telethonkids.org.au; 3 School of Anatomy and Human Biology, The University of Western Australia, 35 Stirling Highway, Crawley WA 6009, Australia; E-Mail: neville.bruce@uwa.edu.au

**Keywords:** cytotoxic drugs, birth defects, female veterinarians, unplanned pregnancy, cohort study, women health

## Abstract

We examined the association of occupational exposure to handling cytotoxic drugs at work with risk of birth defects among a cohort of female veterinarians. This study is a follow up survey of 321 female participants (633 pregnancies) who participated in the Health Risks of Australian Veterinarian project. Data on pregnancies and exposure during each pregnancy was obtained by self-administered mailed questionnaire. Female veterinarians handling cytotoxic drugs during their pregnancy had a two-fold increased risk of birth defects in their offspring (RR = 2.08, 95% CI (1.05–4.15)). Results were consistent in subgroup analysis of those who graduated during the period of 1961 to 1980 (RR = 5.04, 95% CI (1.81, 14.03) and in those working specifically in small and large animal practice. There was no increased risk in the subgroup that graduated after 1980. Women with unplanned pregnancies were more likely to handle cytotoxic drugs on a daily basis (RR = 1.86, 95% CI, 1.00–3.48) and had a higher increased risk of birth defects than those who planned their pregnancies in recent graduates and in those who worked specifically in small animal practice (RR = 2.53, 95% CI, 1.18–5.42). This study suggests that the adverse effects of handling cytotoxic drugs in pregnant women may include an increased risk of birth defects. Pregnancy intention status is an important health behavior and should be considered in prenatal programs.

## 1. Introduction

Toxic drugs are defined as having specific health effects (such as skin rashes, cancer and reproductive effects) and health toxicity at low doses [1]. Most toxic drugs in veterinary medicine are cytotoxic drugs (anti-neoplastic) used to treat animal illnesses such as cancer [1,2]. Many veterinarians treat small companion animals (primarily cats and dogs), but also larger animals such as horses with cytotoxic and other drugs that may be hazardous to humans. Cytotoxic drugs, which are primarily used for oncology treatment, are known to be highly toxic to cells, principally through their action on cell reproduction [2,3]. Many have proved to be carcinogens (C), mutagens (M) or teratogens, which are toxic to reproduction (R), the so-called CMR substances, where no threshold dose for the effect can be identified [4,5]. Cytotoxic drugs are known to be teratogenic and given the mechanisms of their action that can inhibit the growth of tumors by killing actively growing cells, they can also interference with cell division and cell formation in the fetus. Data on therapeutic exposure to these drugs indicate that pregnant women are most susceptible to fetal loss and teratogenicity effects of these agents during the first trimester, a time of rapid cell division and differentiation in the embryo/fetus [6,7].

Occupational exposure to cytotoxic drugs and related waste also presents a possible risk. The reproductive toxicity of cytotoxic drugs has been reported mainly in female nurses working in clinical oncology settings [7,8,9], and in female pharmacists and technicians preparing ready-to-administer cytotoxic drug solutions [10]. The available evidence suggests that occupational exposure to cytotoxic drugs might constitute a risk factor for spontaneous abortion and be related to infertility [7,11,12]. In veterinary practice, exposure to cytotoxic drugs can occur when preparing and administrating these drugs for cancer therapies in animals [13]. Although many safety measures were implemented to reduce worker exposure in the 1980–1990s, continuing research in this area, promoted by the National Institute for Occupational Safety and Health (NIOSH), demonstrates ongoing exposure [14]. Further, a 2010 study [15], assessed the impact of the 2004 NIOSH Alert on hazardous drugs by evaluating potential sources of exposure in the workplace using surface sampling in selected pharmacy and nursing areas, self-maintained exposure diaries and urine and blood samples from various health care workers, and detected contaminated surfaces in all sites.

The limited evidence from human epidemiological studies regarding occupational exposure to handling cytotoxic drugs establishes a need to further examine these associations with birth defects. We conducted a follow-up survey of those female veterinarians who have already participated in a large retrospective cohort study titled “Health Risks of Australian Veterinarians (HRAV)” [16,17,18,19,20,21], to examine additional risk factors such as handling cytotoxic drugs and perceived psychological stress in relation to adverse reproductive outcomes. The present report investigates the risk of birth defects in offspring of female veterinarians working in clinical practice and contrasting those exposed to cytotoxic drugs with those not exposed to these drugs.

## 2. Experimental Design

*Study Design*: This study was conducted in 2005–2006 and was a follow-up survey of the Health Risks of Australian Veterinarians Study (HRAV) conducted in 2002 (Shirangi A. Occupational hazards in veterinary practice and possible effects on reproductive outcomes in female veterinarians [doctoral thesis], School of Population Health, Faculty of Medicine, Dentistry and Health Sciences, The University of Western Australia, 2006) [16,17,18,19,20,21]. The HRAV project was the first complete national retrospective cohort health survey of all graduates from Australian veterinary schools during the 40-year period 1960–2000, and is described in detail elsewhere [16,17,18,19,20,21]. In 2005, previous female participants of the HRAV study who responded to the first survey were re-contacted and invited to participate in a follow-up survey to complete a supplementary questionnaire to address the gaps from the first survey. One of the gaps from the first survey was to examine the risk of birth defects in pregnancies of female veterinarians handling cytotoxic drugs. Questionnaires were mailed to 925 female veterinarians who have already participated in the first survey, with 632 replies (68% response). Of the 632 female veterinarians who participated in this follow-up survey, 220 had never been pregnant and 412 had at least one previous pregnancy. The questionnaire collected detailed information on demographic details, pregnancy status, fertility history, pregnancy intention, menstrual cycle characteristics, working situation and occupational hazards, lifestyle factors, perceived work and general stress, and reproductive history and outcomes. All subjects gave their informed consent for inclusion before they participated in the study. The study was conducted in accordance with the Declaration of Helsinki, and the protocol was approved by the Human Research Ethics Committee of the University of Western Australia (Project Identification RA/4/1/1158 code).

*Exposure Assessment*: Working situation and occupational exposure to handling cytotoxic drugs were assessed in relation to the timeframe of individual pregnancies covering the critical times of vulnerability (3rd–8th week of pregnancy). Respondents answered questions relating to each pregnancy including whether they had worked in veterinary medicine, the job type (clinical, government, academic, drug company, other), clinical role (sole owner, partner, associate, locum), animal practice type (small, mixed, large), work hours and handling of cytotoxic drugs used at work (daily, weekly, rarely, never). Most of toxic drugs used in veterinary clinical practice in Australia are cytotoxic drugs and the female vets have rarely had occupational exposure to other CMR medicinal products. Information was also obtained regarding lifestyle factors in relation to the timeframe of each pregnancy such as marital status (married, single, living with partner), cigarette smoking (numbers/day), caffeine consumption (coffee/cola/tea, cups/day), alcohol intake (units/week), physical activity (hours/week) and sexual activity (times/week). Data on stress levels directly related to the timeframes of individual pregnancies were collected, with questions about work-related stress and stress in general, social support at work and home, and clinically diagnosed anxiety or depression.

*Health Risk Assessment*: Reproductive history was assessed, for each pregnancy, including whether the pregnancy was planned, the number of months trying to become pregnant (time to pregnancy), duration of pregnancy (in weeks), and complications of pregnancy. Reproductive outcomes were assessed from information on live births, stillbirths, terminations (induced abortions), miscarriage (spontaneous abortions), ectopic pregnancy, birth defects, birth weight, sex of child and plurality. Birth defects were defined as a structural or functional abnormality, present at conception or occur before the end of pregnancy and diagnosed by six years of age. Birth defects were coded by staff at the Western Australian Birth Defects Registry (WABDR) according to the British Paediatric Association (BPA) ICD-9 system and without knowledge of the exposure status of the mother.

*Cohort Selection*: Pregnancy was the unit of observation used in this study. Study entry was restricted to women with past singleton pregnancies who were eligible to be assessed for the outcome of birth defects. We excluded the following pregnancies: those where conception began before graduation from veterinary college (*n* = 22), twin pregnancies as we only had information on one twin (*n* = 16); ectopic pregnancies (*n* = 2), those pregnancies current at the time of the survey (*n* = 31); pregnancies that ended in miscarriages (*n* = 115), those women who didn’t work exclusively as clinical practitioners (*n* = 162); those terminations of pregnancies after 20 weeks of gestation without birth defects (*n* = 16) and those with missing data or ‘not sure’ reports of birth defects (*n* = 9). After all these restrictions, out of 1,007 pregnancies (in 412 women), 633 (62.9%) pregnancies in 321 (77.9%) women were included in the analysis.

*Statistical Analysis*: Generalised Estimation Equation (GEE) Modelling by a modified Poisson regression, with robust error variance, was used to estimate relative risk (RRs) and 95% CI for the association between exposure to handling toxic drugs and birth defects [22,23]. We estimated relative risk using glm (generalised linear model) and log-binominal model by a modified Poisson regression model in STATA. The GEE models estimated the effects of independent variables including the main exposures and confounding variables on several types of outcomes, namely dichotomous outcomes, such as presence or absence of birth defects with a logit function, and took into account the potential auto-correlation created by more than one pregnancy for a single woman. The model will also address the limitations from the log-binominal model for the RR where it produces confidence intervals that are narrower than they should be and also the problem of convergence problems. The cRR is the ratio of the probability of the event occurring in the exposed group *versus* a non-exposed group and is a ratio of prevalence rather than incidence. The four adjusted RR models estimated the outcome of all malformations in (a) the total study cohort; (b) a stratified sample of old graduates (1960–1980); (c) a stratified sample of recent graduates (after 1980); (d) a stratified sample of those who worked exclusively in small animal practice. We transpired in 1980 to be able to compare the results from this study with other studies in the literature where the effect of current guidelines on safe handling practices was evaluated before and after 1980s. We also estimated the risk in those who worked in large animal practice, but the crude risk estimate only is presented in the results section due to small numbers and lack of power in full adjustment multivariate analysis. These secondary analyses on stratified samples were undertaken to estimate the risk in even more homogeneous groups and because higher occupational exposures and birth defects were reported in these groups. The following occupational variables were examined as exposures: practice type, handling cytotoxic drugs used at work, years in job and working hours. Occupational exposure to pesticides, anaesthetic gases and x-rays as independent variables were assessed from the first survey and the results for these exposures have previously been reported [19]. For this survey, they were treated as potential confounders.

The final models were adjusted for all exposure variables plus maternal age, smoking, alcohol drinking, marital status, graduation year, work and general stress, and social support from work and home. Inclusion of covariates was based on the magnitudes of regression coefficients and on *a priori* considerations as to the importance of the risk factors. Exposure to handling cytotoxic drugs has been evaluated for the response effects of the daily use and weekly use compared to those who were unexposed (rarely or never used). Interaction effects of factors related to birth defects were examined and the only significant interaction was between cytotoxic drugs and work stress, which was included in the model.

## 3. Results

A total of 633 eligible pregnancies were included in the study of which 59 (9%) had diagnosed birth defects by age six years (Table 1). Cardiovascular defects were the most common type reported, with a prevalence of 2.05%, comprising 25% of all registered birth defects.

**Table 1:** Prevalence of Birth Defects (1960–2005) in Australian Female Veterinarians by Diagnostic Category and British Paediatric Association (BPA) Codes.

Birth Defects (BPA Code)	Number of Defects *	Number of Birth Defect Cases	Proportion of Birth Defect Cases ** (%)	Overall Prevalence (%) ‡
Nervous system defects (74,000–74,299)	6	5	9.61	0.79
Cardiovascular defects (74,500–74,799)	14	13	25.00	2.05
Respiratory system defects (74,800–74,899)	-	-	-	-
Gastro-intestinal defects (74,900–75,199)	3	3	5.77	0.47
Uro-genital defects (75,200–75,399)	12	10	19.23	1.58
Musculo-skeletal defects (75,400–75,699)	13	12	23.08	1.89
Chromosome defects (75,800–75,899)	2	2	3.85	0.31
Other registered defects	8	7	13.46	1.10
Total registrable birth defects	58	52	88.13	8.21
Not registered defects§	10	7	11.86	1.10
Total registered and not registered birth defects	68	59	100	9.32

Notes: * For infants with more than one defect, each defect was counted separately, so, the number of birth defects reported exceeded the number of affected infants. ** Proportions have been calculated based on total registrable birth defects (100%). **‡** Number of birth defects cases/total number of singleton live births and stillbirth pregnancies plus terminations of birth defects before 20 weeks gestation (633 cases) × 10^2^. § Not registered at Western Australian Birth Defects Registry.

Descriptive characteristics of the study population and working situation are shown in Table 2 and Table 3. Survey respondents were born from 1938–1976, with 77% graduating after 1980 with median and mean age of 42 years.

**Table 2:** Demographic, reproductive and life style characteristics of mothers of 633 pregnancies in 321 female veterinarians employed in clinical practice and its association with birth defects (BD).

Characteristic	No. for Eligible Outcomes ^a^	Percent	No. BD Cases	Crude BD Risk	cRR ^b^	95% CI ^c^
**IEmployed in clinical practice **						
**(small, mixed, large)**	633	100	59	0.09		
**Age at the time of**						
**Survey (2005)**						
<40	220	34.76	19	0.09	1.00	
40–49	311	49.13	31	0.10	1.15	0.66–1.99
≥50	102	16.11	9	0.09	1.02	0.47–2.18
**University**						
Queensland	185	29.23	23	0.12	1.00	
Sydney	171	27.01	19	0.11	0.89	0.50–1.58
Melbourne	182	28.75	7	0.04	0.30	0.13–0.70
Murdoch	95	15.01	10	0.10	0.84	0.42–1.70
**Decade of graduation**						
1961–1980	144	22.75	14	0.10	1.00	
1981–1990	306	48.34	30	0.10	1.19	0.66–2.16
1991–2000	183	28.91	15	0.08	1.18	0.59–2.37
**Smoking during pregnancy**						
**Number/Day**						
No	602	95.10	56	0.09	1.00	
<20 cigarette	17	2.69	1	0.06	0.63	0.09–4.31
20+	14	2.21	2	0.14	1.53	0.41–5.68
**Drinking alcohol during pregnancy,**						
**Standard unit/week**						
No	347	54.82	36	0.10	1.00	
0.2–21	286	45.18	23	0.08	0.77	0.47–1.27
**Coffee/Tea/Cola during pregnancy**						
**Cups/Day**						
0	163	25.75	16	0.10	1.00	
<5	400	63.19	36	0.09	0.91	0.53–1.60
5+	70	11.06	7	0.10	1.01	0.43–2.36
**Maternal age**						
≤35	504	79.87	48	0.10	1.00	
>35	127	20.13	11	0.09	0.90	0.48–1.70
**Marital Status during pregnancy**						
Married	574	91.40	56	0.10	1.00	0.11–1.81
Living with partner	45	7.17	2	0.04	0.45	0.17–7.37
Single	9	1.43	1	0.11	1.14	
**Baby Sex**						
Male	325	51.34	29	0.09	1.00	
Female	308	48.66	30	0.10	1.09	0.67–1.77
**Planned Pregnancy**						
Planned	507	80.09	45	0.09	1.00	
Not Planned	126	19.91	14	0.11	1.25	0.70–2.20
**Time to Pregnancy**						
0–3	490	77.41	41	0.08	1.00	
4–12	120	18.96	14	0.12	1.39	0.78–2.47
>12	23	3.63	4	0.17	2.07	0.81–5.31
**Work Stress ^d^**						
None	51	8.06	2	0.04	1.00	
Low	239	37.76	25	0.10	2.66	0.65–10.91
Medium	258	40.76	25	0.10	2.47	0.60–10.11
High	85	13.43	7	0.08	2.10	0.45–9.73
**General stress out of work environment ^e^**						
None	65	10.27	3	0.05	1.00	
Low	326	51.50	35	0.11	2.32	0.73–7.34
Medium	187	29.54	16	0.09	1.85	0.55–6.16
High	55	8.69	5	0.09	1.96	0.49–7.88

**^a^** Total number of singleton live births and stillbirth pregnancies plus terminations of birth defects before 20 weeks gestation; **^b^** Crude Relative Risks estimation using Generalised Estimation Model by a modified Poisson regression with robust error variance; **^c^** Confidence Intervals; **^d^** Work stress includes too much responsibility, schedule is too high, pressed to work too hard, poor relationship with supervisors, *etc*.; **^e^** General stress includes any major events that disturbed you such as loss of job, marriage, serious illness, depression, loss of family member, moving house, relationship failure, *etc*.

Multivariate regression modelling showed that handling cytotoxic drugs during pregnancy was significantly associated with birth defects among all women (Adj.RR = 2.08, 95% CI, 1.05–4.15) and also in stratified analysis of subgroup of small animal practices (Adj.RR = 2.88, 95% CI, 1.37–6.05) (Table 4) and those who worked specifically in large animal practice (Adj.RR = 3.80, 95% CI, 1.07–13.41. Data not shown in the table). In the whole cohort, working in large animal practices had greater risk of birth defects (Adj.RR = 3.42, 95% CI, 1.68–6.92) compared with those working in small animal practice. There was no increased risk of birth defects in those who exposed to cytotoxic drugs in stratified analysis of the subgroup of recent graduates after 1980. However, recent graduates had higher increased risk of birth defects where it is estimated based on different practice type. Including the pregnancy history variables in the model indicated some interesting results. Women with unplanned pregnancies were more likely to handle cytotoxic drugs on a daily basis (RR = 1.86, 95% CI, 1.00–3.48) and had a higher increased risk of birth defects than those who planned their pregnancies particularly in recent graduates. The risk of birth defect with unplanned pregnancy was significant in those who worked specifically in small animal practice (RR = 2.53, 95% CI, 1.18–5.42). Offspring of women who took 6–12 months and >12 months to become pregnant also seems to have an increased risk of birth defects as shown in Table 1, compared with women taking <3 months to fall pregnant. The result was consistent in multiple regression-adjusted model (data not shown).

**Table 3:** Prevalence and crude relative risks estimation (cRR) with 95% confidence intervals (CI) of birth defects (BD) by occupational hazards during pregnancy in 633 pregnancies in 321 female veterinarians employed in clinical practice.

	No. for Eligible Outcomes ^a^	Percent	No. BD Cases	Crude BD Risk	cRR ^b^	95% CI ^c^
**Clinical Practice type**						
Small	394	62.24	34	0.09	1.00	
Mixed	213	33.65	17	0.08	0.92	0.52–1.61
Large	26	4.11	8	0.31	**3.56**	**1.84–6.90**
**Clinical Role**						
Sole owner	102	16.11	9	0.09	1.00	
Partner	117	18.48	15	0.13	1.45	0.66–3.17
Associate	336	53.08	31	0.09	1.04	0.51–2.12
Locum	78	12.32	4	0.05	0.58	0.18–1.81
**Handling of toxic drugs (used at work)**						
Never/Rarely	537	84.83	46	0.08	1.00	
Weekly	43	6.79	4	0.09	1.08	0.40–2.87
Daily	53	8.37	9	0.17	**1.98**	**1.02–3.82**
**Working hours/week**						
<35	333	52.61	34	0.10	1.00	
35–45	205	32.39	12	0.06	0.57	0.30–1.08
>45	95	15.01	13	0.14	1.34	0.73–2.43
**Years in job**						
<6	38	6.00	3	0.08	1.00	
6.01–9	49	7.74	3	0.06	0.77	0.16–3.63
>9	546	86.26	53	0.10	1.23	0.40–3.75

**^a^** Total number of singleton live births and stillbirth pregnancies plus terminations of birth defects before 20 weeks gestation; **^b^** Crude Relative Risks estimation using Generalised Estimation Model by a modified Poisson regression with robust error variance; **^c^** Confidence Intervals.

**Table 4:** Adjusted Relative Risk estimation using Generalised Estimation Model by a modified Poisson regression (RR and 95% confidence intervals) for Birth Defects (BD) due to selected occupational hazards during pregnancy and pregnancy intentions in 633 pregnancies in 321 female veterinarians employed in clinical practice.

	BD ^1^			BD ^2^			BD ^3^			BD ^4^		
**Occupational hazards**	**Adj. RR**	**95% CI**	***P***	**Adj. RR**	**95% CI**	***p***	**Adj. RR**	**95% CI**	***P***	**Adj. RR**	**95% CI**	***P***
**Clinical Practice type**												
Small	1.00			1.00			1.00			-		
Mixed	0.99	0.50–1.91	0.96	0.24	0.38–1.55	0.13	1.09	0.56–2.12	0.79	-		
Large	**3.42**	**1.68–6.92**	**0.001**	2.56	0.62–10.49	0.18	**3.92**	**1.69–9.07**	**0.001**	-		
**Handling of toxic drugs**												
**(used at work)**												
Never/Rarely	1.00			1.00			1.00			1.00		
Weekly	1.39	0.58–3.33	0.45	1.99	0.53–7.39	0.30	0.90	0.29–2.84	0.87	1.03	0.34–3.08	0.95
Daily	**2.08**	**1.05–4.15**	**0.03**	**5.04**	**1.81–14.03**	**0.002**	0.96	0.40–2.25	0.92	**2.88**	**1.37–6.05**	**0.005**
**Years in Job** (full time and part time)												
<6	1.00											
6.01–9	1.03	0.22–4.68	0.96	0.97	0.91–1.04	0.54	1.02	0.97–1.08	0.27	1.03	0.98–1.08	0.16
>9	1.24	0.44–3.49	0.68		continuous		continuous					
**Working hours/week**												
Continues	1.00	0.98–1.02	0.84	1.02	0.99–1.06	0.15	0.99	0.97–1.01	0.56	1.01	0.99–1.03	0.25
**Planned Pregnancy**												
Planned	1.00			1.00			1.00				1.00	
Not Planned	1.23	0.63–2.37	0.53	1.01	0.21–4.82	0.98	1.70	0.85–3.42	0.13	**2.53**	**1.18–5.42**	**0.01**

^1^ All eligible pregnancies (graduated between 1961–2000)—Adjusted for all other variables in the table plus maternal age, smoking, alcohol drinking, marital status, graduation year, work and general stress, and social support from work and home; ^2^ Pregnancies in women who graduated between 1961 and 1980—Adjusted for all other variables in the table; ^3^ Pregnancies in women who graduated between 1981 and 2000—Adjusted for all other variables in the table plus maternal age, smoking, alcohol drinking, marital status; ^4^ Pregnancies in women who worked in small animal clinical practice (graduated between 1961–2000)—Adjusted for all other variable in the table plus maternal age, marital status, and graduation year.

## 4. Discussion

*Main findings*: We found a 2‒5 fold-increased risks of birth defects for occupational exposure in veterinary practice to cytotoxic drugs on a daily basis during pregnancy. This risk was limited in veterinarians who graduated before 1981 suggesting that current guidelines on safe handling of cytotoxic drugs are being followed more carefully by recent graduates. The results on increased risk on the whole cohort was consistent in those who worked in small and large animal practices. The risk of birth defects was elevated in those with unplanned pregnancies and women with unplanned pregnancies were more likely to handle cytotoxic drugs on a daily basis.

*Strengths and limitations*: This study is a follow-up survey from the HRAV survey and the strengths and limitations of the HRAV study have been discussed in details elsewhere [17,18,19]. First, information on exposure was based on self-report. Detailed occupational exposure information collected from a cohort of highly educated women may have minimised this issue. In addition, previous surveys have supported the use of exposure data from retrospective self-reports [24,25], so effect of recall bias usually tends to be small. Moreover, information on personal habits, lifetime working histories, and reproductive history can be obtained only through questionnaire-based studies.

It is important whether control measures were adequate or protective devices were used during the relevant exposure times. We had no information about the control measures and personal protective equipment (PPE) such as use of hood and glove in regard to handling cytotoxic drug use in this study, but the results from this study on recent graduates suggest that current guidelines on safe handling of cytotoxic drugs are being followed more carefully by recent graduates and this is consistent with many studies assessing this risk after 1980s [26]. We also lacked any information about types of cytotoxic drugs in this study, but there is no evidence in literature to show a differential effect of each type of cytotoxic drug on pregnancy outcomes.

Second, there is a possible selection bias due to loss of follow up (about 20%) of those who participated in original survey, but didn’t participate in this follow up survey. The results indicate that those who didn’t participate in this follow up survey were older as 77% of participants from this survey graduated after 1980 compared to 72% in the original survey. The results also indicate that those who didn’t work in clinical practice (9%) were less likely to participate in this follow up survey as compared to 15% from the original survey and this non-clinical group was not in the cohort inclusion criteria for this study. We reduced the risk of selection bias by including all abnormalities where the pregnancy was terminated, present at birth and malformations diagnosed by the age of six years. Third, we were unable to analyse the data by specific malformations due to small sample size and the loss of statistical power. Further studies investigating the risk of specific birth defects are needed and require larger sample size.

Our study also had several strengths such as; (a) using a specific homogenous occupational group of those who worked in clinical practice with possible intensity in exposure and easier adjustment for covariates and using the comparison group of unexposed pregnant women from those who worked in clinical practice, but unexposed; (b) its sample size represents all types of clinical practice and from all ages in Australia suggesting that our study was generally representative of female veterinarians; (c) allowing the assessment of the exposure during pregnancy including the 3rd–8th weeks of pregnancy; (d) exploring the effect of exposures on potential dose-response relationships using partial quantification of differences according to durations of exposure (per day or per week), but not a more complete quantification that included the average intensities of exposures; (e) performing an explorative stratifying analyses in more homogeneous groups such as small animal practice, large animal practice, recent and old graduates using same comparison groups.

The findings from this population-based analysis of no association between risk of birth defects of unknown etiology and advancing maternal age is consistent with another population-based analysis in British Columbia published in Lancet [27] and also other studies. The findings from this population-based study also indicate that only 5% being current smoker, which is consistent with the results from the original HRAV survey [16] and other studies in Australia [28], and in United States [29,30]. All these results provide evidence of validity and reliability of the data collected from this highly educated women and will provide the recall accurately and will reduce response bias.

*Interpretations*: Limited research has been conducted to explore the risk of birth defects in offspring of women who are exposed to handling cytotoxic drugs and the majority of these studies have focused on health care workers (nurses and pharmacists). Six studies have investigated this topic [8,9,31,32,33,34]. A study that looked at birth defects restricted to exposure after 1985 did not detect an association between work on an oncology ward and malformations, but observed an increased number of malformations among a subset of oncology nurses who reported cytotoxic therapy (adjusted OR = 5.1 (95% CI: 1.1–23.6) [31]. A case control study conducted after 1985 reported a non-significant risk OR = 5 (95% CI: 0.8 to 34.0) for cleft palate only in working women who had workplace exposure to cytotoxic drugs during the first trimester of pregnancy [34]. A Canadian study using a sample before 1985 reported twice the expected rate of congenital defects in 152 pregnancies of physicians and nurses who had administered cytotoxic drugs during the first month of pregnancy compared to women in the same community (eight defects compared with four expected defects, *p* = 0.05) which is consistent with the results for this study [32]. A Finnish case control study with exposure period of 1973 to 1979 found an odds ratio of 4.7 (*p* = 0.02, eight cases), for malformations in the offspring of nurses handling cytotoxic drugs more frequently during pregnancy and an OR of 2.1 (ns, 11 cases) to women who handled cytotoxic drugs less frequently than once a week [9]. There were no clusters of specific malformations in these studies. We found no increased birth defect risk in those who graduated after 1980 that is consistent with some studies as mentioned above. This is good news for Australian veterinarians working with this type of drugs and is consistent with the changes in the preparation and administration of cytotoxic drugs and the risk of acute and long-term toxic effects in health care workers, which have been declined over the past 20 years [26]. However, detectable levels of cytotoxic drugs have been reported in the urine of pharmacists, pharmacy technicians, nurses and workers actively handling these compounds working in a hospital [35]. The increased risk of birth defects due to exposure to handling cytotoxic drugs in those who graduated for the period of 1961–1980 will be important for those developing countries with similar work conditions to the Australian veterinarians during 1961–1980.

There were also a high proportion of unplanned pregnancies and its association with exposure to cytotoxic drugs and risk of birth defects indicates a high possibility of harmful occupational exposures during early stages of pregnancy. Consistent with literature [36,37] our findings suggest that pregnancy intention status is a key determinant of pregnancy-related health behavior. Longer times to get pregnant seemed to be associated with increased risk of birth defects in our data. This finding is consistent with previous studies indicating its association with other adverse pregnancy outcomes [38,39]. The etiological mechanisms associated with subfertility and adverse pregnancy outcomes need further investigation.

## 5. Conclusions

Consistent with the animal and human data from previous survey, the results from this study suggest that the adverse effects of handling cytotoxic drugs in pregnant women may include an increased risk of birth defects. The results for recent graduates indicate the safe handling of cytotoxic drugs in the current veterinary practice in Australia. Women with unplanned pregnancies were more likely to handle cytotoxic drugs on a daily basis and had a higher increased risk of birth defects than those who planned their pregnancies in recent graduates and in small animal practice. The findings from this study are important in clinical practice when providers are caring for female veterinarians and other health care workers with similar exposures. Pregnancy intention status is important health behavior and should be considered in prenatal programs.
